# Reforms to improve reproducibility and quality must be coordinated across the research ecosystem: the view from the UKRN Local Network Leads

**DOI:** 10.1186/s13104-022-05949-w

**Published:** 2022-02-15

**Authors:** Suzanne L. K. Stewart, Charlotte R. Pennington, Gonçalo R. da Silva, Nick Ballou, Jessica Butler, Zoltan Dienes, Caroline Jay, Stephanie Rossit, Anna Samara, U. K. Reproducibility Network (UKRN) Local Network Leads

**Affiliations:** 1 School of Psychology, University of Chester, Parkgate Road, Chester, CH1 4BJ UK; 2 Institute of Health & Neurodevelopment, School of Psychology, College of Health & Life Sciences, Aston University, Birmingham, B4 7ET UK; 3 School of Biological Sciences, Queen’s University Belfast, 19 Chlorine Gardens, Belfast, BT9 5DL UK; 4 Game AI Group, Queen Mary University of London, Mile End Road, London, E1 4NS UK; 5 Centre for Health Data Science, University of Aberdeen, Aberdeen, AB25 2ZD UK; 6 School of Psychology, University of Sussex, Falmer, Brighton, BN1 9QH UK; 7 Department of Computer Science, University of Manchester, Oxford Road, Manchester, M13 9PL UK; 8 School of Psychology, University of East Anglia, Norwich Research Park, Norwich, Norfolk, NR4 7TJ UK; 9 Centre for Thinking & Learning, Institute for Lifecourse Development, School of Human Sciences, University of Greenwich, Old Royal Naval College, Park Row, Greenwich, London, SE10 9LS UK

**Keywords:** Science and Technology Committee, Integrity, Reproducibility, Transparency, Open research, Open scholarship, Research infrastructure, UK Reproducibility Network

## Abstract

Many disciplines are facing a “reproducibility crisis”, which has precipitated much discussion about how to improve research integrity, reproducibility, and transparency. A unified effort across all sectors, levels, and stages of the research ecosystem is needed to coordinate goals and reforms that focus on open and transparent research practices. Promoting a more positive incentive culture for all ecosystem members is also paramount. In this commentary, we—the Local Network Leads of the UK Reproducibility Network—outline our response to the UK House of Commons Science and Technology Committee’s inquiry on research integrity and reproducibility. We argue that coordinated change is needed to create (1) a positive research culture, (2) a unified stance on improving research quality, (3) common foundations for open and transparent research practice, and (4) the routinisation of this practice. For each of these areas, we outline the roles that individuals, institutions, funders, publishers, and Government can play in shaping the research ecosystem. Working together, these constituent members must also partner with sectoral and coordinating organisations to produce effective and long-lasting reforms that are fit-for-purpose and future-proof. These efforts will strengthen research quality and create research capable of generating far-reaching applications with a sustained impact on society.

## Introduction

There has been increasing scrutiny of the reproducibility and replicability of published research [1–6], two fundamental principles which cultivate the credibility, applicability, and societal impact of findings across all research fields [2]. Accordingly, in July 2021, the UK House of Commons Science and Technology Committee launched an inquiry into research integrity and reproducibility, stating that while “Government policy has focused on the overall theme of ‘Research Integrity’,…the specific issue of reproducible research has been overlooked” [7]. This commentary outlines our response to this inquiry on behalf of the UK Reproducibility Network’s (UKRN) Local Network Leads. The UKRN is a peer-led consortium which aims to ensure that the UK remains a centre for world-leading research [8]. Within its wider advocacy and training work to improve research integrity and reproducibility, the UKRN connects local researchers’ networks, formal university and research institute members, and stakeholder organisations such as funders, publishers, and policymakers [9].

The research ecosystem must seize this opportunity for coordinated change. Reforms will only be effective, though, if ecosystem members identify common goals and agree on a shared path towards them. We argue that the primary goal should be to improve research quality. A critical mechanism for improving quality is reinforcing and enhancing the openness and transparency of research practice, of which reproducibility and replicability are constituent parts. Consequently, higher quality research would be capable of generating greater societal application and impact [10–12]. The difficult question for all of us in the research ecosystem is, “How do we coordinate our efforts to build a foundation of open and transparent research practice that strengthens research quality?” Here, we attempt to answer this question by outlining the coordinated actions that key research ecosystem members (individual researchers, institutions, funders, publishers, and Government) can take.

## Main text

### Coordinating a positive culture of open and transparent research practice

Rather than an intention to mislead, research of low quality and poor integrity stems from a lack of awareness of and inadequate training in rigorous techniques and methodologies [13] and the habitual use of poor practices encouraged within a system that strongly incentivises quantity over quality [5, 14, 15]. A national Government committee on research integrity should, therefore, focus on positive actions and incentives that support local and national progress towards adopting open and transparent research (OaTR) practice, coordinating with institutions to improve working cultures. Relentless pressure to publish and acquire grant funding is commonplace [14], as is the resulting detriment to researchers’ wellbeing [5, 16]. This pressure is counter-productive for two reasons. First, individuals thrive when they feel supported, recognised for effort rather than achievements, and trusted with autonomy [14]. Second, pressure to publish incentivises closed and opaque research shortcuts that increase the volume of outputs, but which, simultaneously, harm research quality [15].

Instead, institutions should reward and encourage OaTR through their incentive structures [2, 17], for example, through their hiring, induction, probation, promotion, workload, and professional development policies and frameworks (e.g., adopting the Résumé for Researchers [18, 19]). Government can, in turn, incentivise institutions by requiring evidence that reforms have been appropriately implemented. Such policy changes need clear coordination with Equality, Diversity, and Inclusion frameworks [17].

National policies that highlight the benefits of OaTR can guide these coordinated efforts. Government should fully execute its commitment to OaTR [20], as outlined in its Research and Development Roadmap [21], with a specific national policy that incentivises positive, concrete actions and which mirrors effective reforms from other nations. For example, France [22] and the Netherlands [23] have produced systematic and concrete plans for progressing OaTR practice. Indeed, Government can set incentives for individuals, institutions, funders, and publishers to increase engagement with OaTR practice through its Higher Education policies, its funding arms, and its own institutions that conduct research and have in-house ethics and governance processes. Such cultural change will be fostered more successfully through mutually reinforced, coordinated incentive mechanisms rather than strict mandates [24].

Similarly, funders can strategically prioritise calls for meta-research and replication projects [24]. Such initiatives would serve four key purposes: (1) demonstrating to the research community that such areas are valued and important, (2) providing new data about effective improvements to research practice to support evidence-based actions, (3) incentivising individuals and institutions to adopt OaTR practice and replication work, and (4) shifting incentive structures to reward these activities.

The UK Government already recognises through UK Research and Innovation that open access outputs are valuable [25, 26]. This positive cultural change should now be followed by a coordinated, across-the-board effort by publishers to support open access policies and publishing platforms. Funders, institutions (through subscriptions), and individuals (through targeted outlets) can all incentivise publishers to broaden and improve open access policies as well as other avenues that elevate OaTR practice such as pre-registration; Registered Reports [27, 28]; and mandates for sharing data [29], materials, and code [30].

### Coordinating a unified stance for open and transparent research practice

Institutions, guided by sectoral organisations such as Universities UK [31], should coordinate and adopt common policies, guidance, and training for monitoring and improving reproducibility, openness, and quality. For example, UKRN Institutional Leads have worked with Local Network Leads to produce a series of common statements [32] for use by the sector on topics such as research transparency. This collective and collaborative sectoral approach should be informed by the voices of grassroots researchers.

Institutions, Government, and others (e.g., Industry) should coordinate the inclusion of OaTR practice into their research ethics and governance processes. This should take a flexible approach which recognises that open and transparent practice varies by research area and, therefore, input from individual researchers and coordinating organisations such as UKRN is necessary to ensure that updated processes are sensible, executable, and compliant with other relevant frameworks (e.g., funder mandates; legal frameworks; data protection).

### Coordinating the foundations for open and transparent research practice

Institutions, funders [33, 34], and publishers should improve research infrastructure, including coordinated, cross-sector development and/or maintenance of databases, digital storage, servers, software, repositories, and various researcher-led initiatives. Collaboration with individuals and coordinating organisations (e.g., UKRN) would ensure that infrastructure is fit-for-purpose, is interoperable, and avoids duplication.

All members of the research ecosystem should understand the role of knowledge in building a strong foundation for open and transparent research practice. Thus, institutions should recognise the diverse range of specialists who make specific contributions to research openness and transparency [35]. This includes (but is not limited to) data managers, research software engineers, statisticians, laboratory managers, technicians, and compliance officers. To sustain and integrate commitments to improving OaTR practice, institutions should establish core-funded positions for these roles with clear routes for career progression and promotion. Funders should support such roles in their schemes, and publishers should promote creditorship to recognise the contributions of these key individuals [36].

Building a strong foundational knowledge of the practices that promote research quality is paramount for individual researchers, publishers, funders, and Government. Thus, all sectors of the research ecosystem should coordinate and mutually reinforce accessible professional development in OaTR practice (e.g., the UK Data Service’s Learning Hub [37]). Publishers should support or provide training and infrastructure [38] related to the publishing of outputs, including data management, licensing, and digital object identifiers. Similarly, funders should provide accessible training on OaTR practices that they require or encourage. This should be systematically reviewed and updated to reflect ongoing developments. Individual researchers should also be supported and incentivised by all others in the research ecosystem to engage in continuous professional development in OaTR practice, including a focus on the digital skills and infrastructure that facilitate such practice [35]. Individuals must take responsibility to ensure their knowledge and skills remain current. However, this is conditional upon broader cultural changes, including institutions promoting and providing the necessary time for continuous professional development of research skills for individuals at all career stages, in addition to employing specialists.

In turn, individuals have a responsibility to integrate OaTR practice into their teaching and training of students as well as junior researchers whom they manage (see the Framework for Open & Reproducible Research Training [39]). Institutions share this responsibility and can support the longevity of OaTR mentoring by coordinating training, positive incentive structures, infrastructure, and policies for workload and promotion.

### Coordinating the routinisation of open and transparent research practice

A coordinated effort will ensure that OaTR practice becomes routine. All members of the research ecosystem can lead particular actions, while reinforcing others, to embed openness and transparency into the everyday practice of research [2].

Individuals must integrate OaTR practice from the beginning of the research process, including in following relevant ethics and governance processes and in applying for and securing research funding. Individuals should be encouraged to use research infrastructure (e.g., software) and publishing routes (e.g., Registered Reports) that support open and transparent practice throughout the research workflow.

Funders should require the inclusion of planned OaTR practices in funding applications. Applicants should demonstrate whether and how they will share (for example) research data, original materials and protocols, software and code, research workflows, and pre- and post-publication outputs. Depending on career stage, applicants can also be asked to demonstrate a track record of verifiable OaTR practices or professional development plans to achieve this. Additionally, funders should require confirmation that OaTR practices have been followed in funded projects (e.g., in final reports or via Researchfish [40]). Tracking and pooling locations of shared data and other intermediate outputs would allow funders to build searchable databases of available products/outputs and resources that can efficiently support future research and the development of new tools and infrastructure. This would be an advancement on current requirements to simply share data and provide accessible outputs because documented, curated open data and outputs would also become part of the research infrastructure (e.g., the UK Data Service).

Publishers should publish rigorous replication studies [24] alongside tutorials on research processes that can help individuals, institutions, and others improve their own work [41]. As other actions to expand the adoption of OaTR practice take hold, interest in such outputs would continue to increase; hence, for-profit publishers would be implicitly incentivised by changes in supply-and-demand. Furthermore, digital word-limit-free submission formats that promote full and detailed disclosure of methodology, analytical decisions, and pilot work would encourage researchers to fully communicate essential information for reproducibility and transparency, strengthening research quality. Moreover, editorial policies that centre openness and transparency, including systematically checking for compliance with open and transparent practices, should be developed [42]. If sharing data is required, mandated compliance checks can ensure that data are openly accessible both before and after publication (see *American Journal of Political Science* [43]). Such checks can include statistical and analysis code reviews as appropriate [42]. Another area is systematically basing the review and selection of outputs on methodological rigour, openness, and transparency (as indicators of quality) rather than the novelty or nature of findings.

Government, publishers, funders, institutions, and individuals should recognise the value of distributed laboratory networks and collaborative team science in relevant disciplines as models of OaTR practice [44, 45]. Such large-scale collaborations have huge and untapped potential for producing impactful, reproducible, and reliable research findings, and for effectively pooling resources to minimise research waste [45]. Government and funders should incentivise such work through financial support.

## Outlook

As active researchers, we recognise the challenges and the far-reaching opportunities associated with committing to OaTR practice in the context of broader cultural changes. The burden of such changes must not rest primarily on individual researchers. Researchers’ behaviours are a response to the structure and incentives of the ecosystem in which they work; thus, it is imperative that other stakeholders such as institutions, funders, publishers, and Government work with and for individuals [46]. To do so, all research ecosystem members must progress concrete actions, such as those that we suggest here, or risk perpetuating a cycle of discussion where little changes and research quality stagnates or deteriorates [47]. This approach should be collaborative, taking advantage of the interconnected nature of the UK Higher Education system and the existence of coordinating organisations such as UKRN.

## Data Availability

This manuscript is associated with a response from the UKRN Local Network Leads to the House of Commons Science and Technology Committee Inquiry on Reproducibility and Research Integrity.

## Abbreviations

UKRN: UK Reproducibility Network
OaTR: Open and transparent research
